# Removal of biliary casts following orthotopic liver transplantation using a 2.5-mm ultra-fine choledochoscope via the 10-French T-tube tract

**DOI:** 10.1055/a-2839-9542

**Published:** 2026-04-15

**Authors:** Hongzhan Zhang, Ming Zhang, Kai Zhang, Donghai Zhuang

**Affiliations:** 1 Department of Hepatobiliary Surgery, Shandong Provincial Third Hospital, Shandong University, Jinan, China

Bile cast (BC) syndrome is a rare complication of orthotopic liver transplantation (OLT), occurring in up to 4–18% of OLT recipients, but is associated with morbidity, graft failure, the need for re-transplantation, and mortality 1 . For patients with BCs after duct-to-duct anastomosis, endoscopic retrograde cholangiopancreatography (ERCP) is the first-line treatment; however, those who fail often require percutaneous transhepatic biliary drainage or retransplantation 2 . Here, we report, for the first time, a case of successful removal of BCs using a 2.5 mm-diameter ultra-fine choledochoscope via the 10 Fr percutaneous T-tube tract after OLT.

**Fig. 1:**
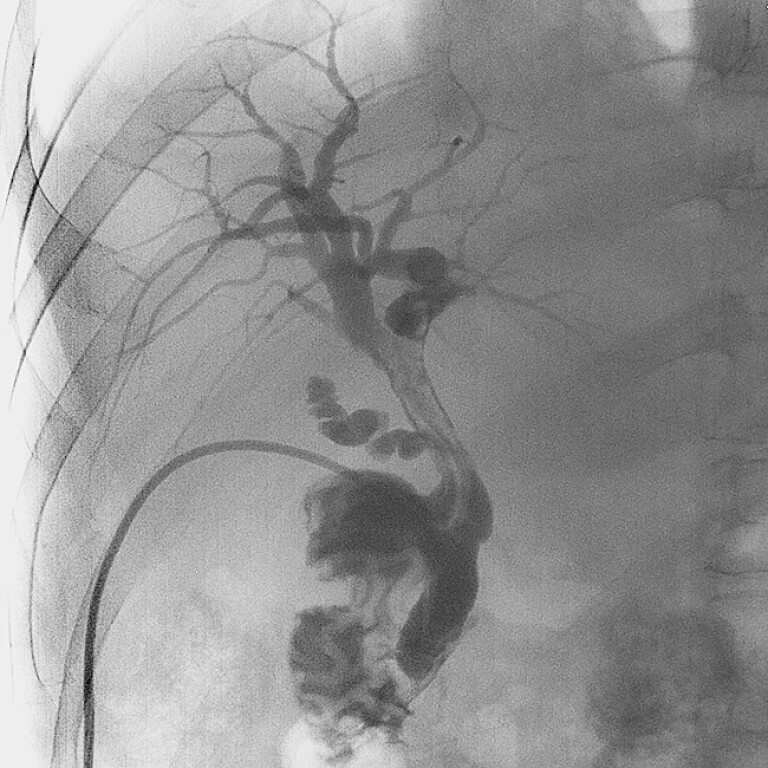
Preoperative T-tube cholangiography revealed diffuse filling defects in the extrahepatic duct, extending into both hepatic ducts.

**Fig. 2:**
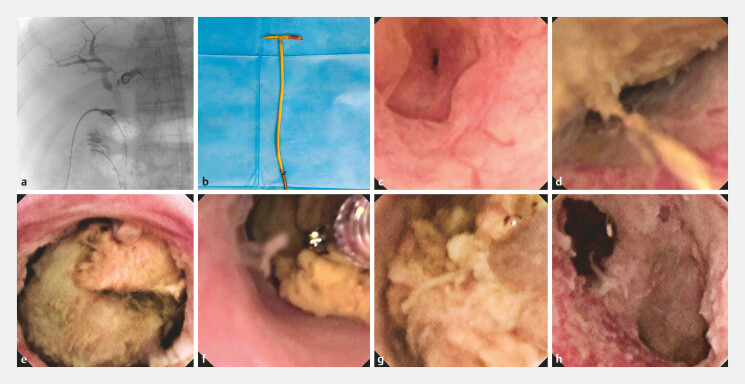
The procedure of removing BCs following orthotopic liver transplantation using an ultra-fine choledochoscope via the percutaneous T-tube tract. **a** Under X-ray fluoroscopy, a guide wire was inserted through the T-tube into the duodenal cavity. **b** Complete removal of the 10-Fr T-tube. **c** Cholangioscopy revealed no abnormalities at the end of the common bile duct. **d** BCs were observed above the bile duct anastomosis. **e** Under direct vision of the choledochoscope, a biopsy forceps was used to pull the BCs into the bile duct below the anastomosis site. **f** BCs were extracted using a biopsy forceps. **g** BCs were extracted using a stone removal basket. **h** Inflammatory changes in the bile duct mucosa were observed in the hepatic portal region, accompanied by necrosis of the biliary duct epithelium. BC, biliary cast.

**Fig. 3:**
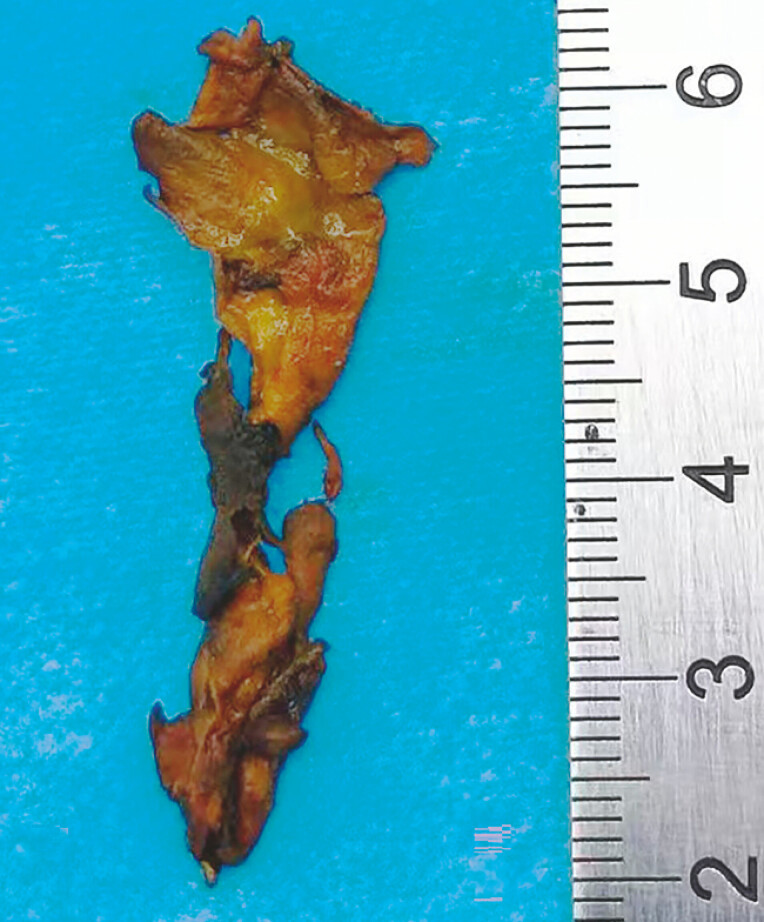
The fragments of the biliary casts were removed using an ultra-fine choledochoscope.

**Fig. 4:**
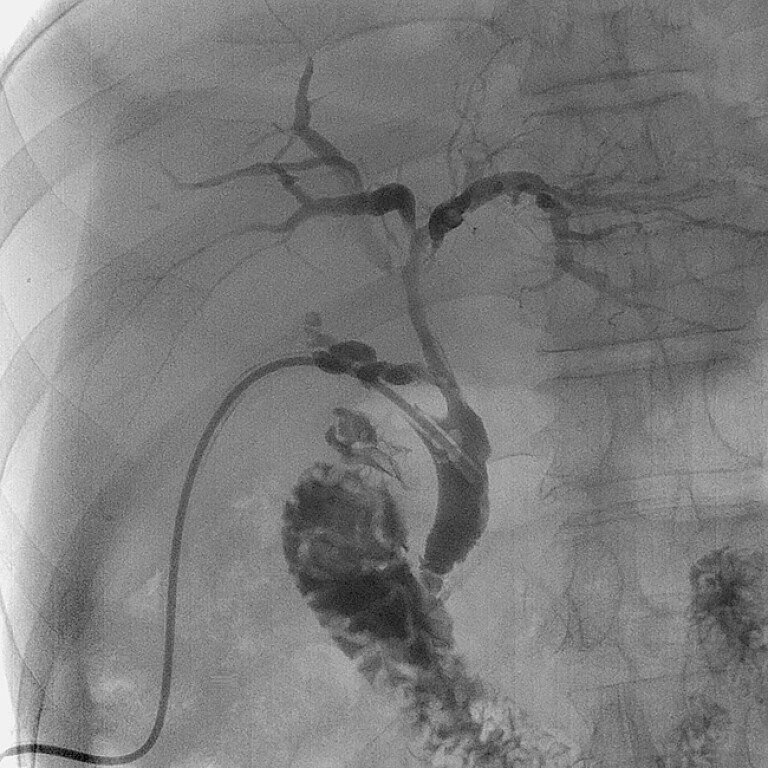
Four days postoperatively, biliary drainage tube cholangiography revealed no evident filling defects or strictures in the intrahepatic and extrahepatic bile ducts.

A 56-year-old man, who underwent OLT 54 days prior due to liver cirrhosis with acute liver failure, had a 10-Fr T-tube indwelled below the anastomotic site during the procedure. In the past 10 days, he began to exhibit jaundice, and bile drainage decreased significantly. T-tube cholangiography revealed diffuse filling defects in the extrahepatic duct, extending into both hepatic ducts ( Fig. 1 ). Comprehensive treatment failed to produce satisfactory outcomes. The diagnosis of BCs was confirmed, and they were successfully removed using a novel short-length (40 cm) ultra-fine (2.5 mm) choledochoscope in combination with one biopsy forceps and a stone removal basket via the percutaneous T-tube tract without dilation ( Fig. 2 and Fig. 3 , Video 1 ). Four days postoperatively, biliary drainage tube cholangiography showed no obvious filling defects or strictures in the intrahepatic and extrahepatic bile ducts ( Fig. 4 ). The patient recovered well and the biliary drainage tube was successfully removed. No BCs recurred during the 6-month follow-up period.

Removal of biliary casts following orthotopic liver transplantation using a 2.5-mm ultra-fine choledochoscope via the 10-Fr T-tube tract.Video 1

The application of a 2.5 mm rubber T-tube following OLT can significantly reduce the incidence of anastomotic strictures, especially in patients with a bile duct calibre mismatch and a diameter of less than 7 mm 3 4 . Ultra-fine choledochoscopy should be considered the preferred approach for managing biliary casts after OLT, as it can provide a precise diagnosis and successful removal of BCs through a 10-Fr percutaneous T-tube tract, avoiding general anesthesia and ERCP-related complications.

Endoscopy_UCTN_Code_TTT_1AR_2AH Endoscopy_UCTN_Code_TTT_1AR_2AL
